# Central Dental Association of Northern New Jersey

**Published:** 1887-04

**Authors:** 


### CENTRAL DENTAL ASSOCIATION OF NORTHERN NEW JERSEY.

Regular meeting, Thursday evening, January 20th, 1887, the President, Dr. B. F. Luckey, in the chair.

Dr. A. W. Harlan, of Chicago, read a paper entitled "Surgical and Therapeutic Treatment of Caries and Necrosis of the Alveolar Process in Man." (See page 169.)

### DISCUSSION.

*Dr. C. S. Stockton*—I have been much pleased and gratified with the paper that has been read. It is true that a large proportion of the diseases incident to necrosis are due to dead pulps in teeth. I have a case now, in which, with the exception of a left central incisor, every one of the ten anterior teeth in the upper jaw is dead. Fluid injected at the second bicuspid on the right side would issue from four or five different openings.

I think destruction of the pulp one of the most important matters that we should guard against. The essayist has very clearly expressed the difficulty met in the removal of this tissue—which should always be done—and I know of no better way of filling the pulp chamber and canal than that which he has described, except that I always use eucalyptus for the wiping out of the canal. I find that the gutta-percha with which I fill the canal is more easily sent home after having used that. I find per-oxide of hydrogen a very excellent preparation indeed, so much so that I have come to the conclusion which, a few years ago, I almost denounced my friend Dr. Atkinson and some others for advising, that you can remove a dead pulp, and at the same sitting fill the tooth and root permanently, and dismiss your patient without fear of subsequent trouble. I know of no agent that is of more value to the dentist than this. When you use it faithfully you know that you have thoroughly disinfected all the parts.

In this matter of bridge-work, which has almost revolutionized the practice of dentistry, our friend, Dr. Truman, was not far from the truth the other evening, when he denounced the driving up of the bands too far around the root of a tooth.

*Dr. W. H. Atkinson*—I desire to congratulate Dr. Harlan upon his growth. How long have we been privileged to hear such papers as we have heard to-night ?

He has spoken of things that are very mixed, and very ambiguous, and very brilliant. It takes a master to discriminate between the alternations of generations of the fore-steps in the growth of the various kinds of seeds that are presented, because each has to follow the type of its own order, which is not manifest until the series is completed. He has given us some very recent nominations and some very old fogy nominations. He has not defined what he meant by caries, as differentiated from necrosis. Caries is superficial necrosis, every time. The old idea of necrosis is that it is death of the molecular conditions of the tissue that is extended over a considerable territory. Does that considerable territory die all at once ? No. There is first an inhibition of the neural currents, then of the vascular currents, and inflammatory action being set up the line or periphery of disturbance of the vessels and nerves that preside over the territory in that neighborhood comes in contact with the healthy tissue and further inflammation is set up, and the line of demarkation is extended. That was the excuse for adopting that abomination, expectant treatment, until the disease had produced the extensive destruction. In medicine or surgery, as in morals, right the wrong action the moment you know it to be wrong. But be careful that you know what you are about. Take Davy Crockett's plan; be sure you are right, then go ahead.

Where is the limit between the living tissue and the dead ? It is dead only in degree. We have not studied these questions of nutrition deeply enough to enable us to do any more than examine mass presentments; we cannot get a clean grip of just where the diseased condition in its lowest expression, or in its earliest exhibition, manifests itself, begins and ends, in order that we may discriminate when we cut into healthy territory, and determine whether the tissue is dead or dying. "Dying thou shalt die," as the book says, meaning that the life principle shall be taken seriatim. The extant pathology stands like a black beast in the way of our diagnosis. But for the misapprehension resulting from it, we would have a better perception of the line of demarkation between healthy and diseased territory, and would know exactly what to do to correct the trouble. Until we understand the series of construction of the tissues, organs and systems, we will not be able to discriminate the retrogression in the nutrient activity that constitutes the disease under consideration.

Unfortunately, whenever we see necrosis everybody thinks there is bone involved. Bone, as bone, never dies. It dies as a molecular mass—as soft tissue. It is the bone corpuscle, the protoplasm in the bone corpuscle, that is the seat of death at first. Nutrition always takes place in soft tissue, always in protoplasmic mass. The deterioration of the tissue has its territory right there in this protoplasmic mass, and in its inception is evidently expressed at that point, and it then extends by reason of the arrest or reversal of the nutrient current. It depends upon how many of those little ponds, with their connections in the canaliculi, are involved, to discriminate how much territory is involved.

How shall we remedy it ? In every case where I find marked periostitis I would open up the cavity and peel off the periosteum until I came to where it has a firm and healthy attachment, and then I would bur away all the dead bone until I get fresh blood. I would not put in a cotton tent. Be sure you have all the dying territory cut away and obtain a clean, fresh surface. Disinfect so that there shall be an aseptic condition present, and then bring the tissues together and leave it to nature. Make the tissues come together in such a way that there will be a pocket formed, into which the serum that makes the first coagulum may be wept. This embryonic tissue, so formed, is differentiated into nerve tissue, muscle tissue and bone tissue. If the cavity is so large that the resilience or stiffness of the walls is not sufficient to coaptate the surfaces so as to form a pocket for holding the serum, then you should use a sponge-graft of a size that will fit the space exactly; or, if you please, make it a little larger, and the serum that is wept out will permeate the trabeculæ of the sponge. Do not put in a drain tube, because in doing so you confess that you are not certain that you have gotten all the diseased tissue out.

Suppose we do make a little mistake in following the line I have indicated; do you suppose the inflammatory process will be set up throughout the entire territory ? No. In such a case cut the sponge down until you come to the territory where you know there has been new growth, then repeat the operation on a smaller scale, with a smaller bit of sponge, and your operation will have been completed. You need not remove that which has already "taken." If we have a sufficient amount of territory, and so shaped as to allow the clot to be formed, then the result will be just such as we obtain in the sponge-graft. When the sides of the sponge-graft and tissue do not "take," and the homunculus does not come up into the pocket and fill it, if you pull a little on the sponge you will see the blood start. You know then that there has been serum wept out and coagulum formed. That is the exact equivalent of the blood seen in a chicken's egg. If there is a little bit of territory that has not been able to receive the pabulum, take a pair of scissors and clip it out, going down until you see fresh blood ooze out, then repeat the operation with a smaller piece of sponge. First disinfect the territory with bi-chloride of mercury, one part to one thousand of water, then insert your sponge. The bi-chloride of mercury in this strength is not an escharotic—it is simply a coagulant. That coagulation is the fibrilation of lymph, and is equivalent to the fibrilation that occurs in the incubation of the chicken's egg. When this tissue has been perfected it is so like the original structure that I no longer call it scar tissue; I call it reproduced tissue.

The paper is chock full of misapprehension all the way through, by reason of the adherence of the author to the old text-book doctrines, and his inability or unwillingness to accept the latest interpretation. But there are so many sharp fellows among us now that it is almost impossible for one swamp-angel of error or ignorance to escape.

*Dr. G. A. Mills*—A recent case in my practice illustrates Dr. Harlan's theory of treating pulpless teeth by medication and immediately filling them. My usual practice is to complete the operation, including medication and filling, at one sitting. If I have any doubt in a case of fistulous opening, feeling that the patient is liable to any disturbance, I tap the alveolus in the territory of the apex of the root, which insures it absolutely against any unpleasant disturbance. This has been my practice for years, following the teachings of Dr. Atkinson.

*Dr. R. Finley Hunt*—I do not feel exactly qualified to criticise this paper in all its details, nor in general. I only wish to make one remark, which I made on a former occasion, and which is based on the opinion that I have stated, that medicine never cures. My opinion is that, as a general rule, these local troubles are the result of constitutional or systemic derangement, determined towards a particular locality where the symptoms are manifested.

The treatment that Dr. Harlan recommends is excellent, because it tends towards the point of removing the local cause of the trouble. My belief has been, for a long time past, that with a proper prophylactic treatment all our local diseases would cease, and we should have no trouble with the teeth. In the opening of his paper, Dr. Harlan gave us the idea that the habits or environments of a certain class of teeth were productive of very serious injuries of the character which he has described to-night. Here is, I think, where many great troubles in the human race originate, as exhibited in the decay of the teeth and the affections of the oral cavity, including both the hard and the soft tissues. The prophylactic treatment case would be a general systemic treatment, mainly through hygiene. When I say hygiene, I include all those agents and accessories which go to make up a perfectly healthy human being. I could enlarge upon that more fully to-night, but inasmuch as I have been requested by Dr. Watkins to read a paper before this society next May, I have concluded that I would select as my subject something bearing on these points, and at that time I hope to present my views to your society in a better manner than I am able to do to-night.

*Dr. Atkinson*—With regard to the remedial agents to be used in these cases, there is a great variety to choose from; but if you will look closely to their chemical constitution you will find that they are almost isomeric, and that their value depends upon their holding oxygen, as one of their equivalents, with a loose grip—as $H_2O_2$, $H_2O$., and $CO_2$ which is carbonic di-oxide, or carbonic acid gas. It has no affinity for the tissues whatever, and is therefore no poison. Where the combustion is complete, $CO_2$ may escape in bubbles with the pus, and has been mistaken for oxygen by those who do not take into account the chemical metamorphosis that takes place. These agents are less specific than they have been supposed to be. We might enumerate turpentine, eucalyptol, sanitas, oil of cloves, eugenol, carvacrol, carbolic acid, creosote, hydro-napthol, resorcin, and many others. Eucalyptus, eucalyptol, eugenol and carvacrol, are simply turpentines with an extra equivalent of oxygen loosely held.

Diseased actions are many times producers of ptomaines or dead protoplasmic bodies, which are sometimes very poisonous, at other times negative, or food. They are efforts at making ashes. Instead of being oxides they are disoxides—alkaloids—the chemical equivalency of which we are just beginning to comprehend.

*President Luckey*—If there is nothing further to be offered in the discussion of this paper we will pass it and call upon Dr. Marvin, who has a paper to read to us to-night.

Dr. C. A. Marvin then read a paper, substantially the same as that read before the Second District Dental Society, at its January meeting. (See INDEPENDENT PRACTITIONER for February.)

DISCUSSION.

*Dr. R. Finley Hunt*—I would like to say a few words in connection with this paper, because of the differences that have arisen in regard to the International Medical Congress. I may be confused to-night, or the paper may be mixed; it certainly seems to me that one or both are very much mystified. I cannot agree with the author of the paper in all the positions taken, nor in the conclusions arrived at. I do not propose to go into any statement of this disagreement. As the paper states, and as we know from our reading, opposite positions were taken up in a former paper, to which this is supposed to be a reply. When I came to New York, the feeling was running high between two factions of the dental profession, and steps were taken which, in my opinion, were calculated to continue the contention. Last night, however, somewhat to my astonishment, although I had some intimation of what was going to take place, after we had been hospitably entertained by the First District Society of New York, speeches were made by two gentlemen, one of whom sits on my right (Dr. Kingsley), and who was considered, I believe, to be the head and front of one of the factions—I call them so simply because they have been so named—and the other gentleman was on my left (Dr. Allport), and I see his pleasant countenance now before me, a gentleman who is intimately connected with the section on oral and dental surgery in the International Medical Congress which is to be held in September. The speeches that were made, and the ideas and sentiments and intentions that were conveyed, seemed to me to be a sort of revelation of a new state of affairs. Last night, at the close of the meeting, I was not able to form an opinion upon the subject; it has been a great deal in my thoughts to-day, and the conclusion I have drawn is that the occurrences of last night constituted a treaty of peace between the two parties, which would inure to the benefit of science, and, if I understood correctly, to the benefit of our profession. I must say, Mr. President, that I feel grateful for this, because I never like to see differences of opinion carried to the extent that these seemed to have been, and with such apparent good grounds on both sides, because the arguments of each of the gentlemen are good and forcible. I suppose that one or the other must be wrong. But it seemed to me last night that the difference was practically settled. The gentleman from Chicago, my esteemed friend of many years' standing, announced his intention, and we must give him credit for sincerity, to advocate with all his might and vigor, and he has a great deal, the assembling of an International Dental Congress in this country at some future day. My friend on my right, whom I highly esteem also, although he is not a friend of so long standing as the other, announced his intention, and the intention of those for whom he spoke, of giving all the aid and assistance in his power towards the success of the section of oral and dental surgery in the International Medical Congress. Thinking it over, I thought this was a healing of a breach that threatened to give great trouble to our profession. At the same time, I must say here that the speeches I heard and the healing of the breach, have not altered my views of the situation in the slightest degree. But I am disposed always to keep my individual opinions in the back-ground, when their promulgation might interfere with the general good and welfare, therefore I abstain from saying anything on this paper, except that either the paper or myself must be somewhat mixed. I arose only to express the hope that what was done last night will receive the sanction, so far as it possibly can be given, of the gentlemen on both sides of the question. I hope that this paper that has been read to-night will give rise to no discussion as a discussion, because the consideration of it at this time will tend to interrupt the pleasant relations now existing between my friend on my right and my friend before me.

*Dr. W. H. Dwinelle*—Nevertheless, Mr. President, inasmuch as the paper which has been read to-night, and which we have listened to with a great deal of interest, is an attack upon a paper that was read upon a previous occasion, the author of which paper is here present, I think that in the interests of justice and courtesy we cannot but call upon him to make his rejoinder. I think we all have sufficient confidence in him and in his method of disposing of a difficult subject ; we have sufficient confidence in his courtesy and in his method of dealing with a subject which has never approached so delicate a character as it has to-night, to feel warranted in extending to him the courtesy of a reply. I hope, therefore, that the author of the paper will be called upon.

*Dr. Hunt*—I was fully aware of the apparent injustice that I was doing to my friend Dr. Kingsley. I did not intend to do him any injury, because I am satisfied that he can meet the positions taken in that paper and answer the objections. I have even had the temerity to suppose that I could myself answer and refute many of the positions taken in that paper. But it was simply in the interests of harmony that I made the suggestion, while I laid aside my own desires in relation to the matter. I think it would not be exact and equal justice to give my friend Dr. Marvin the last say, but I was speaking only in the interests of our profession. I have not the slightest objection to hearing any discussion on the subject.

*Dr. Atkinson*—Mr. President, I think that every gentleman who has spoken has seen the aspect of this question which to him was satisfactory. I agree with everything that has been said on both sides of the question, by reason of what occurred last night in New York, and to which reference has been made here. It would be unfair to further agitate this matter; it would imply that what we heard last night was insincerely said, and I hold that, in justice to our own sense of uprightness, we are bound to take the asseverations of last night as final regarding the differences that seem to have existed. Dr. Kingsley said distinctly that he would do all in his power to further the interests that he had been supposed to attack; and, on the other hand, I think it was the general sentiment of the assembly that all would join to make their best effort to see, in coming time, that an International Dental Congress shall be called. Those of us who are old enough will recollect that this proposition is not a new thing. A good many years ago there was a committee appointed for that very purpose, in the American Dental Association.

And I say, as my individual judgment, that Dr. Kingsley never stood on so high moral ground before our body as he did last night. The only implication that can reverse that judgment is that what he said was insincere, and I am sorry for the individual who is willing suspiciously to question the interior working of any man's mind.

*Dr. Allport*—Mr. President, I need not express to you nor to this society my very great admiration of the ability displayed in the paper that has called out this discussion. Whilst there may be, perhaps, in the minds of some, a lurking suspicion of something wrong, I for one do not wish to charge him with it. I am bound to say that I believe him to be sincere in what he said. Yet I do most earnestly believe that the position he has taken is wrong. I do not believe that dentistry is, or ever can become, an independent profession. We derive all knowledge from the collateral sciences, and ours particularly draws from a medical source that which makes our operations intelligent. Take away from them the medical instruction which makes them scientific, and ours becomes nothing but a mechanical pursuit, or an artistic mechanical pursuit. Some medical knowledge is as necessary in our profession or specialty as it is for the making of intelligent operations upon the eye or the ear, or any other part of the body where the operation is surgical, and our ability in this direction must rest upon the foundation of medical intelligence.

In regard to the paper just read to-night, I must say that it is a very able paper, and I think the essayist has answered the arguments of Dr. Kingsley in a very forcible manner. There is no longer, in my judgment, any question as to whether dentistry is a specialty in medicine. It seems to me that no sensible man can deny it, any more than he can deny that any other department of the healing art that deals with a particular part of the human organism is a specialty in medicine.

The question is not as to whether it is a medical specialty, but as to whether we are medical specialists, and it is a mere matter of education and of fact. If we treat the human organism, or any part of it, without having a medical education, we are not medical specialists, any more than is an uneducated corn doctor or a barber. A man who has not the foundation of some medical education is in no sense a medical specialist, although he practices a medical specialty. It requires a medical education to make an intelligent practitioner in any department of the healing art; with that medical knowledge he becomes a medical specialist; without it he is not. I like to be called a specialist, and to have our profession called a medical specialty; but before passing my judgment or my opinion as to whether he who practices it is a medical specialist or not, I feel very much as Mr. Weller did when he said he loved weal pie, especially if he knowed the woman that made it, and was sure it wasn't kittens. If I am to pass any judgment as to whether a man practicing dentistry is a medical specialist or not, I must know the man who practices it, and have some information as to whether or not he knows anything about medicine. If he does not, he is in no sense a medical specialist. I do not mean that a man must have gone through the whole curriculum of a medical college and received its diploma. I simply want a basis of medical knowledge. I do not care where a man gets it, so long as he possesses it and is an intelligent man.

Mr. President, I am reminded that there is something awaiting you up stairs that is much better than anything I can give you, but just a word more. In regard to what occurred last night I would say that I receive it in all sincerity. As those who have been opposed to this section are going to take hold of it and try to make it as interesting and creditable to our profession as possible, I do not believe there is a man engaged in it who will not, when the time comes for the proposed International Dental Congress, be willing to take his place in the ranks and work for the success of that also; but if we wish to make an International Dental Congress a memorable meeting, let us work faithfully to secure a favorable issue for the Medical Congress.

*Dr. N. W. Kingsley*—Mr President and gentlemen, it hardly seems fair that I should be called upon at the last moment to reply to a paper that has been read this evening, reviewing a paper of mine which was never read before this society. I am not aware that there is any one here who possesses sufficient knowledge of my paper to say whether the so-called review touches one of my arguments, and yet, at the last moment, I am forced into a corner, with no opportunity to reply or to state what my position is on this question. Besides, it has been already announced that you are in expectancy of something better up stairs than can be offered here. I cannot do it; it is too late.

*President Luckey*—It was the intention of the chair to extend to Dr. Kingsley all possible courtesy in the way of opportunity to answer the paper and the criticisms, and the chair is very sorry if Dr. Kingsley feels hurt at being crowded out or not having sufficient time. It certainly was not the intention of the chair to show any discourtesy to him, but on the contrary to give him the fullest scope in answering the paper and the statements made therein.

*Dr. Kingsley*—How many minutes will you give me now?

*Dr. Levy*—Ten minutes are sufficient to get to the station.

*President Luckey*—Dr. Kingsley, there are ten minutes left at your disposal.

*Dr. Osmun*—Mr. President, I dislike very much to see the discussion shut off in this manner. Dr. Kingsley ought, in all justice, to have an opportunity to present his views, and if there is any way of getting to Newark to-night without taking the first train, I move you that he have ample time.

*Dr. Kingsley*—Gentlemen, let me appeal to you, then, to earnestly consider the few words that I have to say, because they must be compressed into the briefest possible sentences.

I may say that I agree with everything that has been brought forward in the review read this evening, excepting the attempt to cast a reflection upon the title of President of the New York State Society, which has been used in connection with my paper. It is true that I was so announced in Boston before the paper was read there, but it was not through any suggestion of mine. I am the President of the New York State Society, and I am proud of the honor, but that society is not in any way responsible for my utterances in that paper; nevertheless, I can assure you that the sentiments there expressed are the sentiments of a large majority of the members of the State Society.

Nobody denies that dentistry is a branch of the healing art. Every one who knows anything about it knows that it is a department of the healing art; but the *practice* of dentistry is one thing, and the *practice* of medicine is quite another thing.

The two departments of the healing art have arrived at that stage when they are as distinct and separate from each other as are the army and navy. The army and navy are branches of the general government; they both use powder and guns, and both kill men, but they are entirely distinct organizations; their discipline, arrangements, methods and everything are totally distinct from each other, notwithstanding they have many things in common and have a common purpose, the defense of the government.

Medicine and dentistry are distinctly separate professions, not only practically, but also in their organizations, associations, institutions of education, literature and degree. No abstract theories or sentimental ideas of kinship can blot out the force of these great facts.

I do not use the term medicine in the broad sense of the healing art, but entirely as it is understood by the public; when we speak of a medical man we always mean a physician.

During the last three days we have seen in New York the largest gathering of dentists ever known, and I ask if you saw in all that great assemblage a single practicing physician. Was there a single member of the medical profession, practicing medicine, in attendance at those clinics or who cared anything about what was going on there? I think not. Was not that convention, in its debates and its clinics, as separate and distinct from anything that medical men are interested in as though we had nothing in common, notwithstanding the fact that both professions are branches of the healing art?

There is one sentence in my paper that nobody has yet attempted to answer, and I believe it is so pregnant with truth that it is absolutely unanswerable. It contains the pith of the whole matter; and I believe that if you will reflect upon that sentence and realize how much of truth it contains, I will have 90 per cent. of this audience, and of men practicing dentistry, with me on this question. That sentence is this: "If to-day all the medical colleges and the entire medical profession were to be blotted off the face of the earth, the practice of dentistry would not be injured in the least, nor would humanity suffering from diseases of the teeth be one whit the less cared for."

Gentlemen, is that true or not? If it be true that at this day the medical colleges and the medical profession can leave the face of the earth and dentistry go on uninterrupted, is it not an independent profession?

